# Vertigo as One of the Symptoms of Lyme Disease

**DOI:** 10.3390/jcm10132814

**Published:** 2021-06-25

**Authors:** Klaudia Sowula, Joanna Szaleniec, Mateusz Dworak, Maria Przeklasa, Małgorzata Maraj, Piotr Ceranowicz, Jerzy Tomik

**Affiliations:** 1 ENT Department, Faculty of Medicine, Jagiellonian University Medical College, 30-688 Krakow, Poland; sowula.k@gmail.com (K.S.); joanna.szaleniec@uj.edu.pl (J.S.); dworakmateusz90@gmail.com (M.D.); maria.m.przeklasa@gmail.com (M.P.); 2 Department of Physiology, Faculty of Medicine, Jagiellonian University Medical College, 31-531 Krakow, Poland; malgorzata.maraj@doctoral.uj.edu.pl (M.M.); piotr.ceranowicz@uj.edu.pl (P.C.)

**Keywords:** Lyme disease, *Borrelia burgdorferi*, vertigo, dizziness

## Abstract

Objectives: The aim of the study was to evaluate the frequency of vertigo symptoms and potential labyrinth damage in patients with diagnosed Lyme disease (LD). LD can affect the vestibulocochlear nerve, leading to hearing loss and vertigo/dizziness. Material and Methods: The study included a group of 38 patients between the ages of 20 and 77, who were hospitalized due to vertigo/dizziness between 2018 and 2019. All of the patients underwent a detailed medical interview and an otolaryngological and neurological examination, including video electronystagmography (VENG), in addition to audiological and diagnostic tests. Additionally, ELISA and Western blot tests were performed to confirm the diagnosis of LD. Results: In 20 patients (53%), the Romberg trial was positive (*p* < 0.001). The degree of vestibular dysfunction as shown by the VENG test was associated with the rate of hearing loss as confirmed by the Auditory Brainstem Response (ABR) test (*p* = 0.011), and it mainly concerned high-frequency sounds (*p* = 0.014). Conclusion: Vertigo can be a symptom of LD. It is often associated with labyrinth and hearing-organ damage, which can imply that the inner ear or nerve VIII is dysfunctional in the course of this disease. Antibiotic therapy is effective in reducing otoneurological symptoms.

## 1. Introduction

Vertigo is one of the most prevalent symptoms reported by patients, and its occurrence increases with age. Among the most common causes of vertigo are otolaryngological diseases, nervous system diseases and systemic and organ dysfunctions [1]. One alleged group of diseases which can trigger vertigo involves infectious diseases of the nervous system. Many pathogens are said to be in part responsible for inflammation; among them are spirochetes of *Borrelia* as well as other pathogens transmitted by ticks [2]. Lyme disease (LD) is an increasingly common and recognized infectious disease. Infection occurs as a result of a tick bite, a parasite which is a vector of numerous pathogens, e.g., viruses, bacteria and protozoans [3,4]. In Europe, the highest LD incidence is reported in Germany, Austria, Slovenia, Sweden and Poland. [5,6]. The main transmitters of infection are ticks—Ixodes ricinus, in Asia—Ixodes persulcatus, and in North America—Ixodes scapularis and Ixodes pacificus [7,8]. LD manifests itself with many clinical symptoms and depending on the phase, early or late, they relate to skin, joints, the heart and the central nervous system. Neurological manifestation of LD is referred to as Lyme neuroborreliosis (LNB) and occurs in 10–15% of patients. Most frequently, patients with LNB develop mononeural neuritis, meningitis, or cranial nerve palsy (especially of the facial nerve). There are isolated cases of vestibulocochlear nerve damage which presents with sudden deafness, sensorineural hearing loss or vertigo [9]. LNB reflects the capacity of *B. burgdorferi* sensu lato to invade diverse targets within the peripheral and central nervous system and to cause neurological complications weeks to months after infection [10]. Early nervous system involvement is usually manifested by the involvement of cranial and/or peripheral nerves or nerve roots typically associated with lymphocytic meningitis [11], (Table 1).

LNB may affect the abducens nerve, and very rarely the vestibulocochlear nerve, the optic nerve (optic neuritis, papilloedema), the oculomotor system (NN III, IV), the trigeminal nerve and the caudal cranial nerves (NN IX–XII) [12,13]. It is questionable whether isolated damage to the vestibulocochlear nerve occurs in the context of an acute Borrelia infection [14], hence our interest in this subject and attempts to explain these unknowns.

## 2. Aim of Study

The aim of the study was to evaluate the frequency of vertigo symptoms and potential labyrinth damage in patients with diagnosed LD.

## 3. Material and Methods

### 3.1. Patients

The study included 50 patients (35 women and 15 men) with Lyme disease diagnosed at the Infectious Disease Department, University Hospital in Krakow. Mean age at entry was 44.6, SD = 17.0 years (range: 20–77). Then, patients were diagnosed laryngologically in the ENT Department, University Hospital in Krakow between 2018 and 2019. 

All patients participated in the same detailed protocol that included age, gender, presence of tinnitus or/and vertigo, biochemical blood samples, cytomegalovirus (CMV) and Borrelia serological findings. 

Pregnant women, patients with cardiovascular diseases, central nervous system diseases as well as those that had undergone otologic surgeries were excluded from the study. 

The study protocol was reviewed and approved by the Bioethical Committee of Jagiellonian University in Krakow (1072.6120.318.2018).

### 3.2. Methods

The ELISA tests (IgM and IgG classes) were performed in order to detect infection with *Borrelia burgdorferi:* 1. Borrelia IgG Elisa Recombinant Antigen—enzyme immunoassay for the qualitative or quantitative determination of IgG antibodies against Lyme in plasma, serum or cerebrospinal fluid (catalog number BI-21032) from Biomedica, Swiss. 2. Borrelia IgM Elisa Recombinant Antigen—enzyme immunoassay for the qualitative or quantitative determination of IgM antibodies against Lyme in plasma, serum or cerebrospinal fluid (catalog number BI-21042) from Biomedica, Swiss.

If a positive or doubtful test result was obtained, the Western blot confirmation test was conducted in both classes (recomLINE Borrelia IgG/recomLine Borrelia IgM qualitative test (in vitro)) for the detection of IgG and IgM antibodies against *Borrelia burgdorferi* sensu stricto, *B. garinii*, *B. afzelii, B. bavariensis* and *B. spielmanii* in serum and plasma (cat. no. for IgM 4277, cat. no. for IgG 4276) from Mikrogen Diagnostik, Germany).

Humoral response starts with IgM antibodies, which usually appear 2 to 4 weeks after infection. Immunoglobulin G (IgG) antibodies appear in serum 6 weeks after infection, reach their peak levels after 4 to 6 months and are detectable in serum for many years.

### 3.3. Criteria for the Clinical Diagnosis of LD (All Present)

1. Erythema migrans (rash at the site of a tick bite) in the patient’s history.

2. A positive or doubtful ELISA test.

3. A positive Western blot test.

Treatment:

All patients were treated after all tests with an intravenous infusion of Ceftriaxon at a dose of 2 g for a period of 3 weeks.

### 3.4. Audiological and Otoneurological Examination 

In all patients a detailed medical interview was conducted, and all subjects underwent laryngological and neurological tests used as standard when examining patients with vertigo, which included the nystagmus test and the Romberg trial. 

The audiometric evaluation was assessed at the initial study, after 30 days and 6 months by pure-tone average audiometry (PTA) on low (250, 500 and 1000 Hz) and high (2000, 4000 and 6000 Hz) frequencies for both ears. Moreover, each patient had additional auditory brainstem response (ABR) and video electronystagmography (VENG) examinations. The outcome data included PTA of hearing thresholds of 500, 1000, 2000 and 4000 Hz. The frequencies of bone conduction were the same as those of air conduction and were determined by the Madsen^®^ MIDIMATE^®^ 622 audiometer equipped with TDK39^®^ headphones.

The ABR test was performed on an ICS Chartr EP 200 Otometrics device using a 2–4 kHz crackle acoustic stimulus with a duration of 100 µs. A hearing threshold of ≤20 dB nHL for each ear separately was assumed as the correct result. The abnormal result was assessed for individual hearing loss thresholds of 20–40 dB nHL, 40–60 dB nHL and >60 dB nHL. The video electronystagmography (VENG) test was performed on the Aquamatic equipment number 24510244. In the VENG study, the excitability of the labyrinths was assessed in caloric tests, assuming canal paresis—unilateral loss of horizontal semicircular canal function—(CP) ≤ 20% as the norm.

Tinnitus reported by patients was divided into low- (≤4000 Hz) and high-frequency (≥4000 Hz) tinnitus according to how frequently it was reported.

The follow-up examination was performed 6 months after the first examination and treatment, and it included audiometry, ABR and VENG tests.

### 3.5. Statistical Analysis

Quantitative data were presented as mean, standard deviation and range, whereas qualitative data were presented as counts and percentages. Comparison of the rate of positive Romberg trials in the studied sample with its rate in the healthy population was performed using a one-sample exact chi-square test. Analysis of dependencies between two qualitative variables was conducted using an exact Pearson chi-square test. Statistical analysis was performed using IBM SPSS26 for Windows Version 26.0. Armonk, NY, USA: IBM Corp.

## 4. Results

Vertigo was reported by 38/50 (76%) patients; 27 (71%)/(54%) of them were women and 11 (28.9%)/(22%) were men. People of different age groups were included in the study. The biggest group comprised patients between 20 and 30 years of age (Table 2). Vertigo symptoms include a sensation of spinning or a feeling of unstable ground, and these symptoms often coincided in our study (31/38 patients (81.5%)). In 5/38 of patients (13.1%) nystagmus was recorded in the laryngological test. 

Patients reporting vertigo underwent the Romberg test and then the VENG test. In 20 patients (53%) the Romberg test was positive (*p* < 0.001). Abnormalities in the VENG test, such as reduced labyrinth reactivity, was detected in 11 patients (29%), while in five patients canal paresis (CP) was 20–40%, 40–60% in three patients and > 60% in the other three patients. The remaining patients had a VENG test result within the norm. 

Hearing loss and tinnitus were symptoms which frequently accompanied vertigo. Sensorineural hearing loss (SNHL) was confirmed in tonal audiometry and ABR tests, and it was recorded in 11 (28.9%) of the patients—in two patients (5.3%) it was bilateral, and in two other patients it presented with sudden deafness. The association concerning the comparison of hearing loss diagnosed in the ABR test with the degree of vestibular damage recorded in the VENG test was statistically significant (*p* = 0.011) (Table 3.).

Comparing the audiometric test with the degree of labyrinth damage in the VENG test, it is stated that this association was statistically significant for hearing loss concerning high-frequency sounds (*p* = 0.014) (Table 4.). There was no such association for hearing loss concerning low-frequency sounds (*p* = 0.088).

Tinnitus occurred in 29 (76.3%) patients, the majority of whom experienced high-frequency tinnitus. 

The control examination was performed in 30 patients (8 men and 22 women). In the control study, the VENG test conducted 6 months after the treatment showed no labyrinth pathology in two patients, whose CP = 20–40%., whereas the ABR and audiometry tests showed improvement in hearing in three patients (7.9%), in two patients with a primary hearing loss threshold of 20–40 dB nHL and in one patient with a primary hearing loss threshold of 40–60 dB nHL. Hearing improvement in the audiometric test was related to high-tone hearing loss. There was no resolution of tinnitus in any of the patients.

**Figure 1:**
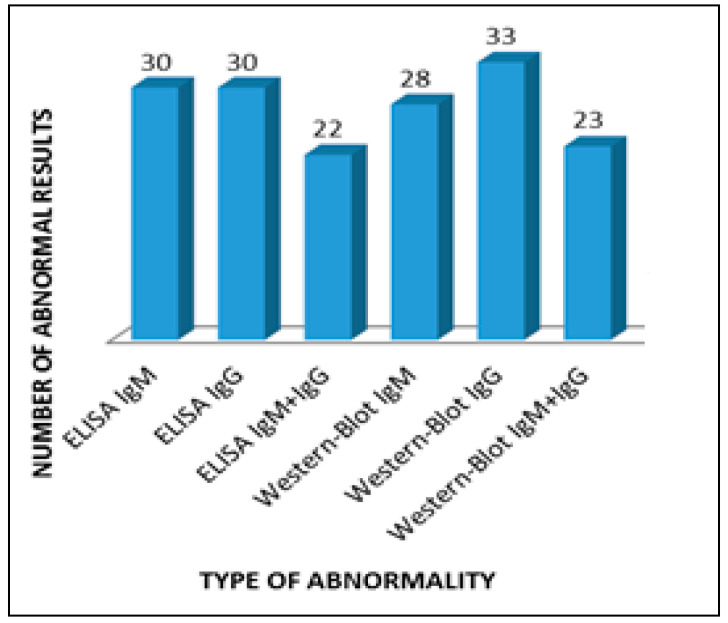
Abnormalities in serological tests in patients with vertigo.

The most numerous group, 33 people (66%), included patients with positive Western blot IgG for borreliosis, and among 30 people (60%) the Western blot IgG test for Lyme disease was positive, which speaks for the presence of vertigo mainly in patients with a chronic form of the disease (Figure 1).

## 5. Discussion

Vertigo is becoming an increasingly prevalent symptom and can mask other diseases not only of the vestibular system but also of a systemic nature. Increasingly, tick-borne illnesses are a potential cause of neurological symptoms reported by patients, including hearing loss, tinnitus, ataxia and vertigo. Balance instability is undoubtedly associated with central and peripheral nervous system dysfunctions. Neurological disorders developed in the course of *B. burgdorferi* infections are a direct effect of spirochetes or its products on nerve cells. What deserves attention is the high tropism of bacteria to the nervous system. It is also suspected that spirochetes have the ability to detect N-acetyl glucosamine, a component of the connective tissue which is essential for growth [15].

In LNB, attention is turned to lymphocyte-T and B-dependent autoreactivity against endogenous neuronal structures. Their presence in connection with the 41 kDa flagellin-enriched protein antigens can induce pro-inflammatory mediators. Inflammatory and angiopathic changes of peripheral nerves can cause injury to axons and consequently lead to peripheral neuropathy [16]. Thus, the most probable underlying cause of neurologic disorders in the course of LNB are vascular changes, and in some irreversible cases, demyelination processes. According to Goldfarb, vascular inflammation can lead to permanent damage to vessels which supply the nerve and in effect lead to axonal neuropathy of different parts of the nervous system [17]. 

In the demonstrated material 38 patients presented with either vertigo or dizziness. Very often, the symptoms coincided with hearing loss and tinnitus, which implies the location of the disease being within the inner ear. Selmani et al. [18] confirmed the occurrence of vertigo in five out of eight patients with LD (62.5%), comparing the results with a group of patients with idiopathic sudden deafness. Ishizaki et al. [19] suggest that vertigo can be the predominant symptom in patients with confirmed LD, its symptoms resembling neuronitis vestibularis in the acute stage. Our own research revealed vestibular abnormality in 11 of 38 patients suffering from LD, with five patients suffering from a slight dysfunction (CP 20%–40%), and only in this group did labyrinth function improve in two patients.

Peltomaa et al. [20] presented eight LD patients with vertigo symptoms, and in all of them, serological tests confirmed increased levels of IgG antibodies, while seven of them had a positive Western blot test result. Six patients presented with rotational vertigo; in two patients, central nervous system damage was recognized in otoneurological tests. Three patients also had a symptom of unilateral sensorineural impairment concomitant with vertigo. Five patients with LD had symptoms resembling Meniere’s disease. 

Sensorineural hearing loss along with vertigo occurred in 11/38 patients in our study. In two cases, it was bilateral hearing loss, and in the next two, it was sudden deafness. This finding agrees with the results of Peltomaa et al. [20]. What is interesting is the observation of the occurrence of more severe hearing loss in the high-frequency range when there was greater damage to the labyrinth. Single cases of hearing loss in patients with vertigo and LD were reported in the literature and concerned unilateral [9] or bilateral [21] hearing loss.

One case of bilateral vestibular hypofunction in the course of LD was presented by van Leeuwen et al. [22]. The patient presented with vertigo and bilateral areflexia in the caloric test. After treatment with Doxycyclin for a period of 14 days, vertigo relented, but the caloric test conducted 6 months later still showed labyrinth damage. 

## 6. Conclusions

The presented study suggests that vertigo can be a symptom of LD. It is frequently connected with labyrinth damage and hearing-organ impairment, which suggests that in the course of this disease the inner ear or nerve VIII is dysfunctional. Antibiotic therapy seems to be effective in reducing otoneurological symptoms, especially in the case of slight labyrinth damage. 

## Figures and Tables

**Table 1:** Classification of Lyme neuroborreliosis (LNB).

**Early LNB** Neurological symptoms for <6 months With manifestations confined to peripheral nervous system (cranial nerves, spinal roots or peripheral nerves) (Bannwarth syndrome) With central nervous system manifestations **Late LNB** Neurological symptoms for more than 6 months With peripheral nervous system manifestations With central nervous system manifestations

**Table 2:** Demographic and clinical characteristics of patients with LD and vertigo.

	Male with LD and Vertigo (*n* = 11)	Female with LD and Vertigo (*n* = 27)
Mean age (years, range)	37.8 (SD = 19.0) 23–77	46.2 (SD = 15.8) 24–70
Age range (%)		
20–30	6 (15.8%)	5 (13.2%)
31–40	1 (2.6%)	9 (23.7%)
41–50	2 (5.2%)	2 (5.2%)
51–60	0	3 (7.9)
61–70	1 (2.6%)	8 (21.1%)
71–80	1 (2.6%)	0
Hearing loss	6 (15.8%)	5 (13.2%)
Sudden deafness	1 (2.6%)	1 (2.6%)
Tinnitus (%)	9 (23.7%)	20 (52.6%)

**Table 3:** The association between hearing loss (recorded in ABR test) and the degree of vestibular damage.

V = 0.54, *p* = 0.011			ABR Hearing Loss		Total
			No	Yes	
VENG (CP)	<20	Sample size	22	5	27
		%	81.5%	45.5%	71.1%
	20–40	Sample size	4	1	5
		%	14.8%	9.1%	13.2%
	40–60	Sample size	1	2	3
		%	3.7%	18.2%	7.9%
	60	Sample size	0	3	3
		%	0.0%	27.3%	7.9%
Total		Sample size	27	11	38
		%	100.0%	100.0%	100.0%

VENG—video electronystagmography; ABR—Auditory Brainstem Response.

**Table 4:** The association between the occurrence of high-tone hearing loss with the degree of labyrinth damage.

V = 0.50, *p* = 0.014			Audiogram High-Tone Hearing Loss		Total
			<20	≥20	
VENG	<20	Sample size	17	10	27
		%	81.0%	58.8%	71.1%
	20–40	Sample size	4	1	5
		%	19.0%	5.9%	13.2%
	40–60	Sample size	0	3	3
		%	0.0%	17.6%	7.9%
	≥60	Sample size	0_a_	3	3
		%	0.0%	17.6%	7.9%
Total		Sample size	21	17	38
		%	100.0%	100.0%	100.0%

## Data Availability

All relevant raw data from the data presented in the manuscript are available from the authors of the study upon request.

## References

[1] Murdin L., Schilder A.G. (2015). Epidemiology of balance symptoms and disorders in the community: A systematic review. Otol. Neurotol..

[2] Langhagen T., Albers L., Heinen F., Straube A., Filippopulos F., Landgraf M.N., Gerstl L., Jahn K., von Kries R. (2015). Period Prevalence of Dizziness and Vertigo in Adolescents. PLoS ONE.

[3] Nelson C.A., Saha S., Kugeler K.J., Delorey M.J., Shankar M.B., Hinckley A.F., Mead P.S. (2015). Incidence of Clinician-Diagnosed Lyme Disease, United States, 2005–2010. Emerg. Infect. Dis..

[4] Franke J., Hildebrandt A., Dorn W. (2013). Exploring gaps in our knowledge on Lyme borreliosis spirochaetes—Updates on complex heterogeneity, ecology, and pathogenicity. Ticks Tick. Borne. Dis..

[5] Stanek G., Wormser G.P., Gray J., Strle F. (2012). Lyme borreliosis. Lancet.

[6] Neuhauser H.K. (2009). Epidemiologie von Schwindelerkrankungen. Nervenarzt.

[7] Paules C.I., Marston H.D., Bloom M.E., Fauci A.S. (2018). Tickborne Diseases—Confronting a Growing Th reat. N. Engl. J. Med..

[8] Patton S.K., Phillips B. (2018). CE: Lyme Disease. Am. J. Nurs..

[9] Józefowicz-Korczyńska M., Zamysłowska-Szmytke E., Piekarska A., Rosiak O. (2019). Vertigo and Severe Balance Instability as Symptoms of Lyme Disease—Literature Review and Case Report. Front. Neurol..

[10] Mygland A., Ljøstad U., Fingerle V., Rupprecht T., Schmutzhard E., Steiner I. (2010). EFNS guidelines on the diagnosis and management of European Lyme neuroborreliosis. Eur. J. Neurol..

[11] Radolf J.D., Strle K., Lemieux J.E., Strle F. (2021). Lyme Disease in Humans. Curr. Issues Mol. Biol..

[12] Oschmann P., Dorndorf W., Hornig C., Schäfer C., Wellensiek H.J., Pflughaupt K.W. (1998). Stages and syndromes of neuroborreliosis. J. Neurol..

[13] Reik L., Steere A.C., Bartenhagen N.H., Shope R.E., Malawista S.E. (1979). Neurologic abnormalities of Lyme disease. Medicine.

[14] Rauer S., Kastenbauer S., Hofmann H., Fingerle V., Huppertz H.I., Hunfeld K.P., Krause A., Ruf B., Dersch R., Consensus Group (2020). Guidelines for diagnosis and treatment in neurology-Lyme neuroborreliosis. Ger. Med Sci. GMS E J..

[15] Farshad-Amacker N., Scheffel H., Frauenfelder T., Alkadhi H. (2013). Brainstem abnormalities and vestibular nerve enhancement in acute neuroborreliosis. Case Rep. BMC Res. Notes.

[16] Ercolini A.M., Miller S.D. (2005). Role of immunologic cross-reactivity in neurological diseases. Neurol. Res..

[17] Goldfarb D., Sataloff R.T. (1994). Lyme disease: A review for the otolaryngologist. Ear Nose Throat J..

[18] Selmani Z., Pyykkö I. (2014). Cochlear and vestibular functional study in patients with sudden deafness an Lyme disease. IJOHNS.

[19] Ishizaki H., Pyykkö I., Nozue M. (1993). Neuroborreliosis in the etiology of vestibular neuronitis. Acta Otolaryngol Suppl..

[20] Peltomaa M., Pyykkö I., Sappälä I., Viitanen L., Viljanen M. (2000). Lyme borreliosis, an etiological factor in sensorineural hearing loss?. Eur. Arch. Otorhinolaryngol..

[21] Huda S., Wieshmann U.C. (2012). Protracted neuroborreliosis--an unusual cause of encephalomyelitis. BMJ Case Rep..

[22] van Leeuwen R.B., van Kooten B., de Cock A.F. (2017). Bilateral vestibular hypofunction and Lyme disease: A causal link?. Acta Neurol. Belg..

